# Insights into Delivering Cross-Cultural Medical Education in the UK and Malaysia

**DOI:** 10.1007/s40670-021-01382-z

**Published:** 2021-09-16

**Authors:** Clare Guilding, Paul Khoo Li Zhi, Sailesh Mohana Krishnan, Paul Stephen Hubbard, Kenneth Scott McKeegan

**Affiliations:** 1 School of Medical Education, Faculty of Medical Sciences, Newcastle University, Newcastle upon Tyne, NE2 4HH UK; 2 University Hospital of North Durham, County Durham and Darlington NHS Trust, Durham, UK; 3 Newcastle University Medicine Malaysia, Gelang Patah, Johor, Malaysia

**Keywords:** Medical education, UK, Malaysia, Cross-cultural, Internationalisation, Clinical pharmacology, Interprofessional education

## Abstract

Newcastle University UK operates an international campus, NUMed, in Malaysia. NUMed delivers the same medical degree programme as in the UK, within a different cultural context. In this paper, medical education faculty and NUMed graduates with experience working in both the UK and Malaysia provide insights into cross-cultural diversity in approaches to learning. Observations from small and large group teaching and approaches to assessment are discussed in relation to students’ cultural backgrounds including previous learning experiences and English language abilities. We provide practice points for educators preparing a diverse range of students to work in global healthcare settings.

## Introduction

### Internationalisation of Higher Education

Driven by the influences of globalisation, higher education institutions (HEIs) are striving to become more international [1–4]. Efforts to internationalise have seen HEIs focus on international staff and student recruitment, increased collaborations and research outputs with international partners, exchanges and mobility for students and staff, and a focus on understanding and improving the internationalisation at home student experience [5–7]. One output of the internationalisation agenda has been the rapid expansion in international branch campuses (IBCs), where one country (home) opens and operates a campus in another (host) [8, 9]. In South East Asia (SEA), internationalisation has been a key strategy in the development of Malaysia’s Higher Education sector in recent years [10–12]. As part of this strategy, Newcastle University in the United Kingdom (UK) was invited to establish a campus specialising in medicine at a newly developed education hub, EduCity@Iskandar Malaysia in the state of Johor [13].

### Context: Medicine at Newcastle

Newcastle Medical School was established in 1834 in the city of Newcastle upon Tyne in the UK. In 2009, Newcastle University established a fully owned IBC in Malaysia, called Newcastle University Medicine Malaysia (NUMed). NUMed offers an undergraduate degree in medicine (MBBS), awarded by Newcastle University. This dual accredited 5-year degree is the first full overseas medical degree programme approved by the UK regulators, the General Medical Council (GMC) and accredited by the Malaysian Medical Council (MMC) [14]. The MBBS curriculum is outcomes based, integrated, and case-led [15, 16]. The same outcomes and assessments are delivered on both sites, with delivery of content similar, yet tailored to the local context. The UK campus (Newcastle) has an intake of approximately 370 students per year, with less than 10% international students. The Malaysian campus (NUMed) has an intake of approximately 150 students per year, with 30–50% international students, mostly from Asia. Faculty at NUMed are a mixture of Malaysian and International staff together with around a quarter of staff seconded from Newcastle University UK. On graduation, MBBS students have the option to apply for provisional registration with the GMC, and for Malaysian citizens additionally the MMC. Graduates need to undergo a period of compulsory internship (called “Foundation” in the UK, “Housemanship” in Malaysia) before they gain full accreditation and licence to work as an independent doctor.

### Context: Authors’ Experience

The authors CG, PSH, and KSM are experienced in teaching and senior management within Newcastle University’s MBBS programme in the UK. KSM (2013–2017) and CG (2017–2020) were, and PSH currently is, seconded to Malaysia as Dean of Academic Affairs, a role that oversees management of all academic elements of the NUMed MBBS programme. The authors PKLZ and SMK were Malaysian MBBS students at NUMed from 2013 to 2018, who undertook placements in the UK during their degree programme. PKLZ worked as a Teaching Fellow at NUMed for a year post-graduation, and both have experience working as doctors in Malaysia and the UK.

The transnational experiences of the authors in teaching, learning, leadership, and management in medical education have enabled us to observe and reflect on cross-cultural diversity in learning and teaching. Observations are naturally influenced by the experiences of our upbringing [17, 18]. For CG, PSH, and KSM, this is from the perspective of being born and educated in the UK, then working within HEIs in the UK until secondment to NUMed. PKLZ and SMK are influenced by being born in Malaysia and educated in Malaysian schools before entry into NUMed. Through collective reflexive discussions, we have sought to make sense of our transnational experiences [18]. Additional insights for this paper were drawn from the literature, colleague observations and expertise, discussions with Newcastle and NUMed students who have experienced cross-campus placements, including a year 2 student exchange programme, and our experiences participating in, establishing, and running transnational placements.

This paper starts with a brief discussion around cultural factors that may influence approaches to learning. We then outline some broad generalisations of diversity in approach to learning of the Newcastle and NUMed students and provide examples of challenges translating Newcastle University’s MBBS curriculum into the Malaysian context. We finish with practice points for educators teaching diverse groups of students within Higher Education (HE).

## Culture and Learning

Culture is all around us: it operates on a personal, local, national, and global level [19]. Current understandings view culture as complex, flexible, and dynamic, arising from cohesive social groupings, as opposed to being a fixed national or ethnic entity that determines an individual’s behaviour [19, 20]. Thus, individuals exist in and are influenced by a multitude of cultural contexts, including their family upbringing, religion, sex, education, occupation, position in society, and nationality. Regarding the national context, in East and South East Asia, there are several countries described as having a Confucian Heritage Culture (CHC) in which individuals share social behaviours including learning approaches, based on Confucian values [21]. Countries said to have CHC include China, Vietnam, Korea, Japan, Singapore, and Malaysia [22, 23]. A body of literature details the different cultural expectations of the student–teacher relationship between “Western” (North American, Western Europe, and Australasia) and CHC learners, and the challenges this poses for students studying within a different cultural context [22–28]. Some of these challenges can be ameliorated through a better understanding, by both educators and students, of cross-cultural differences in academic expectations. We briefly outline some of the theories around dimensions of culture, which illustrate the diverse ways researchers have attempted to understand the impact of culture on the learning experience.

### Individualism and Collectivism

In groups described as collectivist, society is viewed as an extended family, in which everyone has their role to play. The emphasis of behaviour is on actions which benefit the collective group, maintaining social harmony matters more than striving to stand out as an individual and fulfilling one’s own goals [29–31]. Groups or individuals who are more individualistic are driven by their own preferences and needs. There is an emphasis on autonomy, independence, and individual as opposed to group achievement [29–31]. Expectations of levels of support from the teacher and the meaning of groupwork may differ according to a person’s individualism-collectivism orientation [28].

### Hierarchy and Power

Power is the degree to which a person can influence the behaviour and ideas of another. Not all individuals in society are equal, and there is variability in the extent to which unequal distribution of power is accepted or seen as appropriate [32]. Many forms of inequality exist which may be due to differences in experience, social standing, age, or time in a group. The impact these inequalities have on a group dynamic is influenced by societal acceptance of hierarchy. Malaysia is a more hierarchical society than the UK; thus, the expectations of power dynamic in the classroom may be different between students and teachers from the two countries [17].

### High-Context and Low-Context Communication

High and low context relates to how explicit the messages explained in communications are, and the importance of context surrounding communication [33]. In low-context cultures, the message is explained through direct and explicit communication, encoded in written or spoken words. High-context cultures use less direct verbal communication, with greater significance placed on the relationship between individuals, the emotions being transmitted, and interpretation of non-verbal communication [33]. While studies have attempted to classify countries on a low–high context spectrum, generally positioning the UK as low-context and Malaysia as high-context [32, 34], such country-wide classification has been challenged as one example of using nation as a proxy for culture [35]. However, there are variations in context use between individuals, influenced by cultural background, which may manifest in different approaches to communication within the HE setting.

## Cross-Cultural Diversity in Learning and Teaching

NUMed students transition not just to university, but also to a British approach to education; the same is true for international students arriving in the UK [28, 36]. It is in this transition phase to the British education system that differences between Newcastle and NUMed students’ approach to learning were most notable. Below, we provide five observations of diversity which we discuss in relation to the cultural, linguistic, and educational backgrounds of the students. We wish to caveat this section by noting that these are broad generalisations which do not apply to *all* Newcastle or NUMed students, there being a very wide range of inter-individual in addition to international differences [28]. Indeed, the student population at NUMed is diverse, reflecting the major ethnic groups within Malaysia (Malay, Chinese, Indian, and East Malaysians) together with international students from 29 other countries across Asia, Australasia, North America, and Africa.

### 1. Engagement in Answering and Asking Questions in Class

Through our own experience and numerous classroom observations, we observed that Newcastle students were initially more likely than NUMed students to ask and answer questions in class and challenge teachers on the delivered content, particularly in large group lectures. The reserve of some NUMed students may in part be due to English frequently being a second language; early year students especially may lack the experience, proficiency, and thus confidence in speaking English, particularly if pre-university teaching was not in English [36]. Indeed, some NUMed students were taught in Bahasa Melayu, the national language of Malaysia before coming to NUMed (there was period of time where state schools were given autonomy to teach basic sciences in either Bahasa Melayu or English). The reticence of students to speak up in class in a second language is a well-described phenomenon which has been linked to a combination of factors other than language proficiency, including the cultural beliefs around the role of the teacher and learner, the educational background of the students, the comprehensibility of the teacher and teaching strategies, a fear of being unable to be understood, and a fear of loss of face [37–40] (see Table 1).

**Table.1:** The concept of “face”

Face is a term for one’s personal honour and dignity and relates to having a good social standing and being held in esteem by one’s peers. While important in all countries, the emphasis placed on face, and face-saving strategies is particularly apparent in East and South East Asia [32, 41]. In terms of preserving face, studies suggest that students may be reticent to ask questions in case their question is perceived as “stupid” or is a question that others know the answer to, thus demonstrating their “ignorance” [27]. Students may not want to answer questions in case they get them wrong, causing loss of face. In Asia, teachers are frequently held in high regard and openly contradicting them or pointing out something which flags an error in what they’re saying could potentially cause a loss of face for the teacher, which is to be avoided [42]. Equally students may say they have understood a concept so as not to show up the poor teaching or explanation of the ﻿lecture﻿r. In Asia, teachers are frequently held in high regard and openly contradicting them or pointing out something which flags an error in what they’re saying could potentially cause a loss of face for the teacher, which is to be avoided [42]. Equally students may say they have understood a concept so as not to show up the poor teaching or explanation of the ﻿lecturer.	

### 2. Engagement with Lecturers Outside Class

We observed that NUMed students engaged their lecturers more frequently to guide their learning outside the classroom: they were more likely to approach lecturers in-person after class or via email. Some would have done no pre-reading before approaching the lecturers for answers and when prompted to do independent reading around a topic, there were often requests for precise page numbers, or paragraphs for reading. However, others had done in-depth reading and were seeking clarification of complex and detailed material. NUMed students generally sought more detailed guidance on assignments, and where the guidance allowed for variability in structure or content according to the student-selected topic of the assignment, they were more concerned than their Newcastle counterparts about what the “correct” structure was, or what exactly should be included.

Students who seek personal guidance may feel more assured relying on a high-context personal interaction, rather than on the written assignment pack [28]. A desire for more individual guidance may also reflect an orientation towards collectivism [29]. In the more individualistic UK society, children are considered adult when they turn eighteen, leave home, become responsible for themselves, and are expected to be autonomous in learning [43]. Children from societies like Malaysia which hold more collectivistic values remain “children” in the eyes of the older generation for longer than in the UK, so on arrival at university the expectations of guidance and support from parents may transfer to lecturers [44].

From the beginning of an undergraduate degree, UK universities emphasise independence and self-direction in learning [45]. Indeed, a core aim of the Newcastle MBBS curriculum is to produce doctors who “are prepared for on-going professional and personal development and, through this, are able to adapt to future developments in practice”. Time and specific learning outcomes are set aside for self-directed learning in the curriculum; this is viewed as important for adequately preparing students for later clinical years and clinical practice, where postgraduate medical education is largely independent learning [46]. In a dissertation thesis, Braman (1998) noted that self-directed learning is grounded in individualistic values and investigated the cultural dimensions of individualism/collectivism as a factor in self-directed learning readiness (SDLR) [47]. He found that SDLR was strongly associated with individualism and not other variables such as collectivism, age, ethnicity, and gender [47]. A study comparing American and Korean college students identified a more nuanced relationship, but likewise concluded that there is a relationship between these cultural dimensions and self-direction in learning [48]. Each student entering university will be at a different stage of competence in self-directedness in learning, influenced by their cultural and educational backgrounds, and some students will need more support in the move towards independent learning. Educators should take account of this and construct learning activities and study skills training that recognise and address the diverse learning preferences among their student population.

### 3. Engagement with Small Group Interactive and Critical Discussions

A notable area of difference in the early years of the programme was in interactions in seminars and small group discussions which frequently called on students to analyse, evaluate, and debate case scenarios. Newcastle students were more comfortable in giving their opinions and critical in their contributions. As discussed previously, willingness or reluctance to speak up is influenced by a range of factors such as linguistic ability, different understandings of learning, differences in the pre-university experiences of learning, and by personality, for example level of extroversion [49].

In UK schools, students are encouraged to discuss, debate, question, and express their individual opinions as a mode of learning. This form of learning is embedded in a Socratic inquiry and social constructivist approach to education which emphasises learning through discussion, problem solving, and social interaction and is aligned with the teaching methods of case-based learning and small group discussions used in Newcastle’s MBBS programme [50]. It is not uncommon in Malaysia for learning to be based on a Confucian approach, with learning occurring sequentially through the stages of memorising, understanding, applying, and questioning or modifying [50]. Core content in pre-university education in Malaysia is thus often learned through listening to and absorbing information from the “expert” teacher, with repetition and memorisation of set texts understood as the first step on the pathway to critical analysis [21, 26]. NUMed graduates reflecting on the transition to university commented that some had had little exposure to group discussions before university, and one stated they had held the belief they were “here to absorb information from the teacher, rather than to discuss it”.

Studies on UK HE highlight problem solving and critical thinking as a primary goal [51, 52], although the definition of “critical thinking” remains contentious [53, 54]. For international students arriving with a different understanding of the learning process and different experiences of learning, adaptation to a new approach may be challenging [28, 36]. A study of UK university teachers who worked closely with international students noted the students initially struggled in written assignments that involved critical thinking [55]. Difficulties were attributed to an unfamiliarity with UK forms of critical and analytical practice in students’ previous education, together with linguistic difficulties constructing analysis in a second language [55]. Critical thinking is challenging for students from all countries, but, understandably, performance in critical thinking tasks, in particular written and verbal tasks, is more difficult in a second language [39, 53, 56, 57]. Consequently, students in Malaysia may not want to express themselves in seminars until, for example, they are fully confident in their use of English, they have fully understood all aspects of what has been taught (their learning pathway emphasising the importance of knowledge acquisition before critiquing), or until they are comfortable with the mode of teaching and student–teacher power dynamic [20, 21]. Avoiding conflict and disagreement is a commonly held value in Malaysia [58, 59], and the Malaysian authors noted that some NUMed students initially felt uncomfortable with a discursive or debating seminar format and confused by the less hierarchical role of the lecturer.

### 4. Focus on Assessments and Grades

While all students are concerned with assessment, NUMed students more frequently queried what was and was not going to be assessed and were extremely hard working and exam orientated. In Malaysia, education is highly desirable and putting a child through medical school is regarded as investment for the whole family; in Malaysian society, as compared to the UK, there is more emphasis on the younger generations caring and providing for the older generations in later life [60]. University fees are expensive, with a year’s MBBS fees at NUMed costing approximately double the typical yearly salary in Malaysia [61]. The pressure of these expectations means that students work very hard, at times to the detriment of partaking in extracurricular activities. As an example, NUMed had to prohibit students bringing sleeping bags into the library to discourage overnight working. Newcastle students on exchange in Malaysia often noted how hard their counterparts were working, with some commenting that this hard work ethic influenced them positively and that it was something they wanted to take back with them to the UK. Conversely, NUMed students on exchange in the UK noted a better work-life balance among many of their counterparts and reflected this as a positive take-home experience.

The families of NUMed students appeared more involved with and concerned about managing their children’s lives and education than the UK authors had experienced in Newcastle, often requesting details of progress that were unable to be supplied due to data confidentiality. Parents generally paid the university fees and occasionally, difficult conversations arose when there was an expectation that this payment entitled them to know details of the student’s performance. This desire of parents to track progress was likely influenced by the high cost of a medical degree, alongside the fact that parents may assume shared responsibility for their children’s educational journey [44, 62].

NUMed students sometimes appeared less comfortable with topics where there was conflicting evidence: grey areas of science. There was more frequent probing of lecturers for black and white answers, or at least, the “right” answer for the exam. NUMed graduates, reflecting on their desire to know black and white answers in the early years, referred to the importance of grades and ranking for NUMed students, and to their pre-university schooling experiences. In their experience, the Malaysian education system emphasised memorising factual knowledge as the keystone to learning. Memorisation is learning an isolated fact through deliberate effort. For example, reciting facts repetitively and the use of mnemonics, factual knowledge viewed as the foundation for higher thinking and problem solving [21]. However, overemphasis of learning via memorising may inhibit lateral and critical thinking. In 2009, Malaysia participated in the Programme for International Student Assessment (PISA) survey for the first time, results indicating that Malaysian students struggled with problem solving and higher order thinking skills [63, 64]. In response, the Malaysian Ministry of Education introduced the “Malaysia Education Blueprint 2013–2025” emphasising the teaching of higher order thinking skills including critical thinking and problem solving, reflected in less rigid pre-university assessment marking schemes [63]. England has similarly undergone a reform of the A’ level (pre-university) qualifications to encourage the application of higher order thinking skills in the classroom and assessment [65]. It will be of interest to note the impact of these reforms on the newer generations of medical students.

### 5. Professional Approach to Education

NUMed students were very professional in their approach to learning. As a more hierarchical and collectivist society, it is not uncommon for lecturers to be held in very high regard in Malaysia, and students were extremely respectful towards the lecturers and highly motivated in their learning. “Professionalism” of students is assessed each year of the MBBS programme, monitoring behaviours such as attendance and completion of set tasks. NUMed students consistently outperformed UK students on this measure of professionalism. UK faculty who have taught NUMed students have noted how well motivated, prepared, polite, and smartly dressed these students are.

## Translating Newcastle University’s MBBS Curriculum into the Malaysian Context

### Overview of Healthcare and Clinical Placements in the UK and Malaysia

Healthcare in the UK is primarily provided through the National Health Service (NHS). The NHS is a publicly funded government service which is free at point of use. Approximately 11% of the population holds some form of private medical insurance, but this is not usually comprehensive, common exclusions being maternity and mental health cover [66]. Healthcare in Malaysia is organised in two tiers, a publicly funded government service with a nominal consultation fee (1 MYR, ~0.18 GBP, ~0.25 USD) used by around 70% of the population but with only around 45% of the healthcare staff, and private healthcare used by around 30% of the population.

In years 3–5 of the MBBS programme, students are largely on clinical placements throughout the regional medical school. In the UK, the regional medical school extends across most of the North East of England and North Cumbria and includes approximately 300 general practices, 8 acute hospital trusts, and 2 mental health trusts. In Malaysia, the regional medical school extends over the state of Johor and clinical placements occur predominantly in 7 government hospitals and 6 primary care centres, known as Klinik Kesihatan (KK). Malaysia and the UK have a largely similar burden of non-communicable diseases such as ischaemic heart disease, stroke, lung cancer, and chronic obstructive pulmonary diseases [67]. Dementia is more prevalent in the UK which reflects the increasing ageing population in the UK compared to Malaysia. The gap in disease burden is more evident in communicable diseases, as different climates favour different disease vectors. For example, Malaysia’s tropical weather which is hot and humid throughout the year allows vectors such as *Aedes aegypti* and *Anopheles* mosquitos to breed and spread dengue and malaria respectively. In contrast, Lyme disease is predominantly seen in the Northern Highlands in the UK and is almost never heard off within the Malaysian healthcare system.

**Fig. 1:**
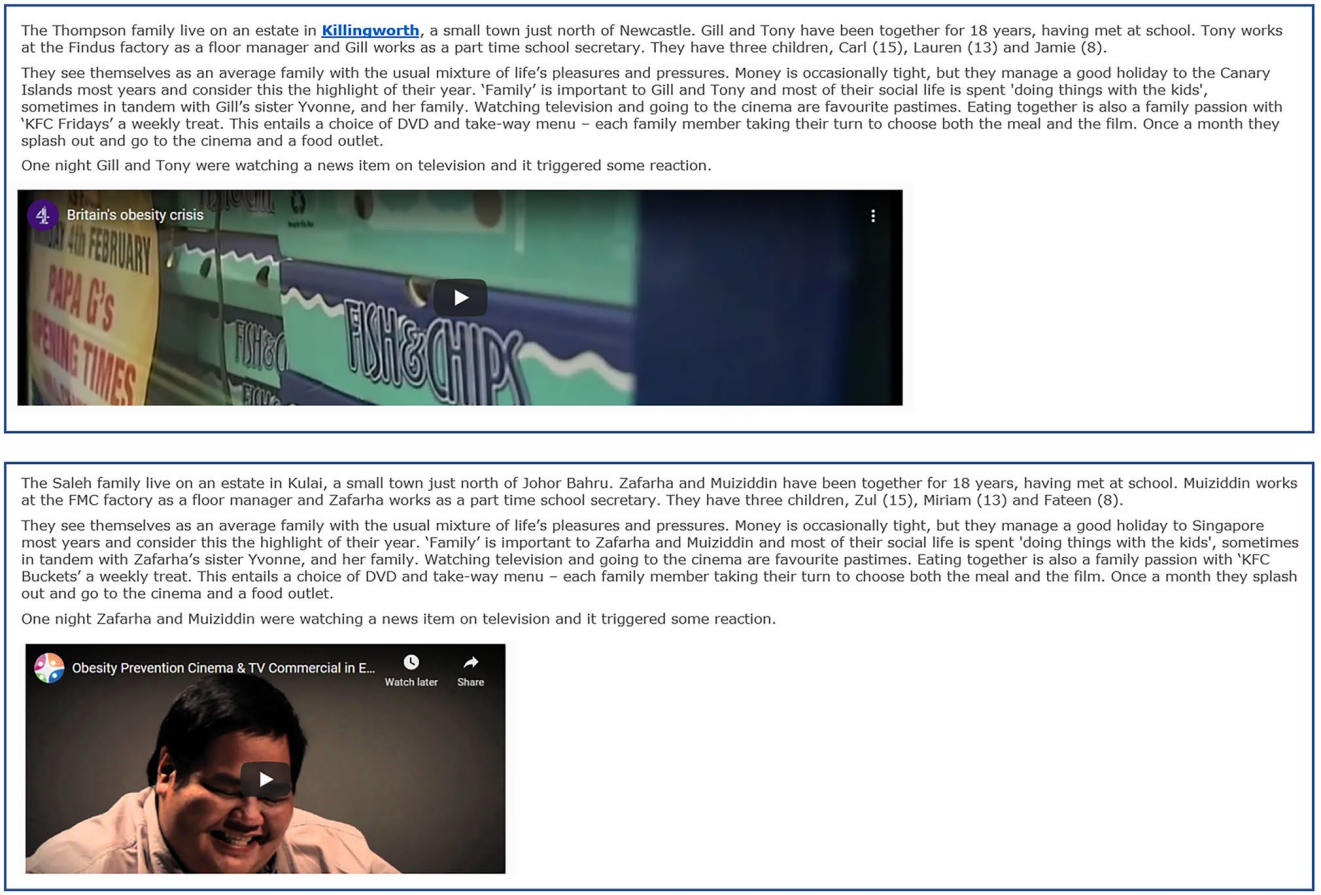
Screenshots from the MBBS Medical Learning Environment (MLE; a custom-made virtual learning environment), illustrating contextualisation of a case to the local setting. The Newcastle (upper) case is based around the Thompson family who live in Killingworth, UK; the video is of Britain’s obesity crisis. The NUMed (lower) case is based around the Saleh family who live in Kulai, Malaysia; the video is about obesity in Malaysia. Students’ MLE is set to show them course content specific for their location

As a medical degree accredited to the professional standards set by the UK and Malaysian Medical Councils, our programme is designed to produce global graduates who are fit to practice as doctors internationally [68]. We have, nevertheless, adapted various components of the curriculum for local context in Malaysia [69]. For example, clinical cases can be set in the local context and pathways of care (see Fig. 1). In clinical years, to align training with workforce needs, more time and emphasis in the curriculum at NUMed is given to specialities that junior doctors in Malaysia but not the UK will be responsible for, such as orthopaedics [68]. Indeed the GMC noted “NUMed is delivering the same curriculum as Newcastle University Medical School in a manner that is sensitive to cultural differences between the UK and Malaysia” as an area working well in their most recent review [14]. However, as a UK university, the curriculum is largely centred around healthcare delivery within the NHS, using UK models of healthcare education and practice and there remain some areas of disparity between the taught curriculum and that observed in clinical practice; we outline a few examples below.

### Paternalistic Versus Shared Decision-Making

When undertaking a medical consultation, there are different broad models for medical decision-making; two of these models are shared decision-making and paternalism [70]. In shared decision-making, there is active participation from both patient and doctor in the decision-making process, and agreement on the decision [71]. In a paternalistic approach, the doctor makes the decision based on what they decide is in the patient’s best interest [72]. The Newcastle curriculum teaches medical students shared decision-making as best practice, while also teaching about contexts within which other decision models may be preferable. However, in clinical placements in Malaysia, students predominantly experience paternalism, and research studies indicate low levels of shared decision-making in Malaysian clinical practice [73–75]. The older generation of Malaysians, the less well educated or those living rurally, may prefer a paternalistic approach, patients looking to the revered doctors to tell them what needs to be done [48, 73]. We have encountered instances of patients being affronted by attempts at shared decision-making, expressing the view that it is the doctor’s job to know what is best and they do not feel qualified to participate in decision-making. A contributing factor to a paternalistic approach is resource limitations [73]. Due to the volume of patients to be seen in the public healthcare system, the consultation often lasts less than 5 min and more than one patient may be in the room at a time, reducing confidentiality [73, 76]. We use this example to illustrate the need to be sensitive to the fact that the healthcare models and practices taught within the Newcastle medical curriculum may not always translate effectively into a different healthcare context.

### Clinical Pharmacology and Therapeutics

Skills and knowledge in clinical pharmacology and therapeutics are assessed in multiple formats throughout the MBBS programme, including in written papers and clinical exams. In their final year, students in the UK and Malaysia sit the Prescribing Safety Assessment (PSA) [77]. The PSA is an assessment of knowledge, judgement, and skills related to prescribing and supervising the use of medicines [78]. The PSA contributes to summative assessment in final year and requires students to be proficient in prescribing and in use of the British National Formulary (BNF). The BNF is the UK pharmaceutical reference book used nationwide by healthcare practitioners for prescribing practice. It is available in book form, online, and is integrated into online prescribing software within the NHS [79]. NUMed graduates highlighted that while the BNF is very relevant in the UK, it is not widely available in hospitals in Malaysia, and is not available online. One NUMed graduate commented that this was their “biggest problem in Housemanship” and they were “lost and didn’t know what source to use”. An additional challenge NUMed students and graduates reported was that in clinical practice doctors frequently used brand names for drugs. On the MBBS programme, we teach generic rather than brand names of drugs, for example ibuprofen rather than Advil or Neurofen. Consequently, NUMed graduates reported not knowing what drugs doctors were prescribing in clinics and wards. This phenomenon varied according to the hospital and was less apparent in large centres with electronic prescribing such as in University Malaya Medical Centre. There is a drive in Malaysia towards increased prescribing of generic drugs and the use of generic drug names on prescriptions, which over time should ameliorate this issue [80].

### Interprofessional Education

Interprofessional education (IPE) is defined “as occasions when members or students of two or more professions learn with, from and about each other to improve collaboration and the quality of care and services” [81]. The General Medical Council has IPE outcomes within their standards, and there is growing evidence that IPE interventions result in changes in behaviour, organisational practice, and benefits to patients [82].

Organising IPE is challenging in all countries due to the logistical challenges delivering sessions to large numbers of students and competing curricula priorities [83–85]. Reports of IPE activities are predominantly from developed countries, with developing countries such as Malaysia incorporating IPE into healthcare education curricula more recently [86–89]. Brock et al. recently reported on the development of an IPE activity for medical and pharmacy students at Monash University, delivered on their Australian and Malaysian campuses [90]. They note the importance of developing context-specific activities, consistent with recommendations from studies that have incorporated Western IPE competency frameworks into South East Asian healthcare education curricula [91, 92].

Large-scale IPE events for medical students operate in Newcastle, but the development of similar learning events in NUMed has been challenging [83, 93]. The NUMed campus is geographically isolated and does not operate other healthcare profession degree programmes. Programmes such as nursing at institutions close to NUMed conduct their courses in Bahasa Melayu, presenting language barriers to integrated learning events. The development of online opportunities for collaborative learning, contextualised to local practice, may provide a novel option for extending the reach of current offerings for both Newcastle and NUMed students [94].

## Supporting the Experience of International Students in HE

Clearer awareness and understanding of cross-cultural perspectives, including preconceptions and expectations about successful education, is an important starting point for both educators and students working transnationally. The UK Quality Assurance Agency and Higher Education Academy (HEA) have published guidance for educators on how to promote intercultural understanding and effective learning, and support international students studying in UK HEIs [28, 45]. HEA guidance on “Engaging home and international students” recommends activities to make explicit the values, knowledge, and experience of teachers and students [28]. These could include discussions around what the outcomes and assessments are and why these are important, how students have been taught previously and how they will be taught now, how students were expected to learn previously and expectations now, and what students can expect from their teachers. There is a body of literature which provides practical suggestions for techniques to integrate and engage students from diverse backgrounds into HE; we outline some techniques we have found relevant in our cross-cultural learning and teaching in Table 2 [27, 28, 95].

**Table.2:** Techniques to integrate and engage students from diverse backgrounds in HE

Area of activity	Suggestion	Rationale
Course induction	Provide a specific and targeted induction for international students during orientation events	At both the UK and Malaysian campuses, international students are provided with a tailored induction session to introduce those students to the local environment A clear induction and overview of expectations, teaching styles, and assessment can aid international students’ integration [96] and overcome some of the academic and cultural challenges of entering into different education systems [97, 98]
Personal tutors	Provide longer term support through a structured personal tutoring system that allows regular one-to-one meetings	In the Newcastle MBBS degree at both campuses, students are assigned academic mentors who are trained members of staff that provide a personalised point of contact to support a student’s personal and academic progress. The academic mentor is stage appropriate: pre-clinical staff support students in years 1 and 2, but on progression to clinical stages students change mentors to core clinical staff attached to the medical school. Long-term support may help to alleviate the “culture shock” international students face, with one-to-one meetings enabling international students to build relations with educators, flattening the hierarchical gap and making it more comfortable to engage in the classroom [99]
Teaching methodology	When teaching, establish a safe and inclusive learning environment: (a) Introduce a collaborative teaching session early in the course to ease international student anxiety and to allow students from different backgrounds to integrate and form social bonds. (b) Set ground rules in discussions and debates allowing students to speak without interruption and encouraging acceptance of a diversity of opinions. (c) Use pair work and collaborative learning tasks to take pressure off individuals responding. (d) Consider collecting questions and answers on paper; or if learning is online, utilise the chat box to gain answers to questions. (e) Ensure online resources contain transcripts/closed captions and edit these to ensure accuracy to reduce issues with misunderstanding of spoken language.	It is well established that a safe learning environment is essential for successful learning [100]. The experiences of the authors and evidence from the literature suggest that international students may find new modes of teaching challenging, related to issues such as language barriers or anxiety over answering questions in class. Creating a safe learning environment and designing teaching approaches that seek to address these differences in previous education experience could help alleviate transitional challenges, and enable better interaction between international and home students [101]
Student-staff interaction	Ensure staff are aware of cross-cultural differences and/or student anxieties and take measures to account for these in student-staff interactions: (a) Make availability outside of the classroom clear. (b) Be explicit about the learning outcomes, the structure of the teaching and the level and form of engagement expected. (c) Speak clearly in face-to-face teaching, facing the front to improve clarity of communication. Encourage staff to be reflective in their approach to teaching international students and accepting of the different learning approaches; for example, not speaking up in class does not equal not-engaging in class.	The differences experienced in the way international students engage with lecturers and the “expert teacher” concept may create tensions between staff and students when staff are used to a more facilitative, or collective approach to teaching and learning [102]. Through acknowledging this, staff can take steps to understand the international student perspective by adjusting both teaching style and student-staff interactions [102]
Assessments	Be aware of the differences in approach to assessment and adjust the way assessments are presented: (a) Explain the pedagogic rationale for the assessment. Link assessment to course learning outcomes and ensure marking criteria are clear, detailed, and explicit. (b) Use clear simple language for all assessment items and guidance, so you are testing the competency of interest, not just English language ability. (c) Provide exemplars of work to aid students in understanding the expected output.	While all students are assessment focussed, there are differences in approach depending on the students’ prior experience, cultural values, and beliefs around assessment. International students may need extra support and guidance to explain the rationale behind different approaches used in their new context

## Future Work

A strength of this paper is that the insights are drawn from multiple perspectives of past and current faculty and students who have experience working in both UK and Malaysia. A limitation is that the paper is based on subjective experience at one university and previous literature, rather than from specific data gained at the university or from experiences across a range of institutions. Future studies could investigate the international student experience of learning and the faculty experience of teaching international students, using focus groups, or by using a validated questionnaire of international students’ cultural and academic experiences [103]. Indeed, we are currently conducting a qualitative analysis of the experiences of Newcastle and NUMed students participating in a year 2 exchange programme, and a quantitative study on differential attainment for medical students from different backgrounds within the medical school.

## Conclusion

A key challenge for health science educators is how to prepare students for their roles in a globalised workplace [104]. In this article, we outline diversity in approaches to learning between students in the UK and Malaysia and discuss the influences of different pre-university learning experiences, different understandings of learning, and the linguistic challenges of studying in a second language. We highlight the need for the curriculum to be sensitive to cultural differences and note some challenges in delivering a shared medical education curriculum in two different countries and healthcare contexts. Ultimately, the course content and modes of delivery of any programme must accommodate different ways of knowing and different expectations, so it is relevant to students from a range of contexts and ensures the contributions of all students to the learning community are valued and recognised [14]. Cross-cultural activities integrated into the start of the programme can improve inter-cultural understanding among students. Similarly, cross-cultural training can improve educators’ cultural self-awareness, knowledge about diverse learners, and aid reflection on the instructional skills needed to best support all students.

## Data Availability

Not applicable.
